# Reply

**DOI:** 10.1054/bjoc.2001.2091

**Published:** 2001-11

**Authors:** E Villa, N De Maria, A Colantoni, M Manno, H Bertani


some instances, favourably influence the course of the disease, but that this, in a consecutive series of HCC patients, happens in a small minority of cases, the treatment being purely palliative in a relatively larger sub-group and of no use in the majority. This, since the variant receptor is to be detected in HbsAg positive patients (Villa et al, 1998), may well have something to do with the fact that we, by now, see mostly patients with HCV-related chronic liver damage and HCC. On the other hand, in four patients development of portal or deep venous thrombosis was observed, which could be considered an expected worsening in patients with HCC, with respect to portal thrombosis, but that could have something to do with the pro-coagulant properties of megestrol at least in the case of deep venous damage (Force et al, 1999). Our experience therefore, albeit limited and uncontrolled, suggests that side effects, particularly on blood coagulation, may not be irrelevant.

In summary, our feeling is that megestrol and the variant oestrogen receptor may be more a step in the understanding of the patho-physiological mechanisms underlying HCC development and progression than a true advance in HCC treatment but we are available to change our mind if new additional data will confirm those reported by Villa et al (2001) and deny our findings. This is also because the tamoxifen story has taught everyone, and us more than others, that preliminary, successful small size trials are to be confirmed by large size prospective randomized placebo-controlled studies before a drug enters routine clinical practice.

### ACKNOWLEDGEMENT

Supported in part by a grant from the Italian Ministry of University and Scientific and Technologic Research (MM06174173–003).

**F Farinati, S Gianni, M De Giorgio and S Fiorentini**

*Department of Surgical and Gastroenterological Sciences, Padua University Policlinic Via Giustiniani 2, 35128, Padua, Italy*

### REFERENCE S

Chao Y, Chan WK, Wang SS, Lai KH, Chi CW, Lin CY, Chan A, Whang-Peng J, Lui WY and Lee SD (1997) Phase II study of megestrol acetate in the treatment of hepatocellular carcinoma. *J Gastroenterol Hepatol* **12**(**4**): 277–281

Colleoni M, Nelli P, Vicario G, Mastrpasqua G and Manente P (1995) Megestrol acetate in unresectable hepatocellular carcinoma. *Tumori* **81**(**5**): 351–353

Farinati F, Salvagnini M, De Maria N, Fornasiero A, Chiaramonte M, Rossaro L and Naccarato R (1999) Unresectable hepatocellular carcinoma: a prospective controlled trial with tamoxifen. *J Hepatol* **11**: 297

Force L, Barrufet P, Herrerasa Z and Bolibar I (1999) Deep venous thrombosis and megestrol in patients with HIV infection. *AIDS* **13**(**11**): 1425–1426

Simonetti RG, Liberati A, Angiolini C and Pagliaro L (1997) Treatment of hepatocellular carcinoma: a systematic review of randomized controlled trials. *Ann Oncol* **8**(**2**): 117–136

Villa E, Dugani A, Moles A, Camellini L, Grottola A, Buttafoco P, Merighi A, Ferretti I, Esposito P, Miglioli L, Bagni A, Troisi R, De Hemptinne B, Praet M, Callea F and Manenti F (1998) Variant liver estrogen receptor transcripts already occur at an early stage of chronic liver disease. *Hepatology* **27**(**4**): 983–988

Villa E, Ferretti I, Grottola A, Buttafoco P, Buono MG, Giannini F, Manno M, Bertani H, Dugani A and Manenti F (2001) Hormonal therapy with megestrol in inoperable hepatocellular carcinoma characterized by variant oestrogen receptors. *Br J Cancer* **84**(**7**): 881–885

### Reply

**Sir,**

Dr Farinati and colleagues have reported an uncontrolled experience with megestrol in the treatment of inoperable hepatocellular carcinoma. Their results were somewhat different from those we have reported in *Br J Cancer* (Villa et al, 2001) and we would like to add some considerations.

Despite the fact that megestrol has a rationale in HCC characterised by both wild-type and variant oestrogen receptors (*v*ERs), as its action is displayed at post-receptorial level (and therefore able to interfere both *v*ERs and wild-type ERs (*wt*ERs), still the natural history of HCC with *wt*ERs is so favourable and the growth speed of the tumours so slow that megestrol or any other antihormonal drug, would not add much in terms of amelioration of prognosis (Villa et al, 2000). The choice of treating with megestrol only patients with *v*ERs was therefore justified by the much more aggressive clinical course of these HCCs, which could allow easier identification of any effect on tumour growth or an improvement in survival. As variant ERs are usually not more than 30% of patients with HCC, the higher percentage of patients with wild-type ERs could obscure a favourable effect of megestrol when this treatment is used in a mixed population.

Furthermore, the uncontrolled design of the study by Farinati et al, could not allow perception of the most relevant finding of our study, i.e. the improvement in survival in treated patients. It was, in fact, already evident from our data that megestrol did not determine regression of tumour mass (except in a few cases) whereas slowing down of tumour growth was remarkable in comparison with untreated patients. This effect was short-lived but sufficient to determine a significant improvement of survival at 1 year (Villa et al, 2001). Certainly, megestrol was not powerful enough to cure HCC, but in these patients with ominous prognosis, a gain of 10–12 months in survival can be considered an achievement. In a few of them, clinical improvement was also accompanied by significant regression of tumour mass which allowed performance of radical treatment (E Villa and V Mazzaferro, personal communication).

Last but not least, the side effects reported by Farinati et al (e.g. portal vein thrombosis, deep vein thrombosis, bleeding etc.) may also spontaneously occur in HCC patients: again the uncontrolled experimental design by Farinati was certainly not suitable for correctly allocating side effects to therapy or to disease. Indeed, in our series increase in appetite and in weight occurred in a remarkable percentage of treated patients and in none of the control. However, as for the vascular complications, deep vein thrombosis occurred in the same proportion in treated and untreated patients.

In conclusion, these considerations underline the need to observe very strict methodological rules when performing therapeutic trials: only a controlled method allows identification and correct allocation of both benefits and side effects.

**E Villa, N De Maria, A Colantoni, M Manno and H Bertani**

### REFERENCES

Villa E, Moles A, Ferretti I, Buttafoco P, Grottola A, Del Bvono MG, De Santis M and Manenti F (2000) Natural history of inoperable hepatocellular carcinoma:

© 2001 Cancer Research Campaign estrogen receptor status in the tumor is the strongest prognostic factor for survival. *Hepatology* **32**: 233–238

Villa E, Ferretti I, Grottola A, Buttafoco P, Del Bvono MG, Giannini F, Mannom, Bertani H, Dugani A and Manenti F (2001) "Hormonal therapy with Megestrol in inoperable hepatocellular carcinoma characterised by variant estrogen receptors: a prospective, randomised, controlled trial" *Br J Cancer* **84**: 881–885

*© 2001 Cancer Research Campaign*

